# Effect of GutGard in the Management of *Helicobacter pylori*: A Randomized Double Blind Placebo Controlled Study

**DOI:** 10.1155/2013/263805

**Published:** 2013-03-27

**Authors:** Sreenivasulu Puram, Hyung Chae Suh, Seung Un Kim, Bharathi Bethapudi, Joshua Allan Joseph, Amit Agarwal, Venkateswarlu Kudiganti

**Affiliations:** ^1^D2L Pharma Research Centre, Bangalore 560 037, India; ^2^NICS, Seoul 135-763, Republic of Korea; ^3^Korea Polytechnic University, Siheung-Si 429-793, Republic of Korea; ^4^Research and Development Centre, Natural Remedies, Plot No.5B, Veerasandra Indl. Area, 19th K.M. Stone, Hosur Road, Electronic City, Bangalore, Karnataka 560 100, India; ^5^Anasuya Ayurveda Centre, Bangalore 560 050, India

## Abstract

A randomized, double blind placebo controlled study was conducted to evaluate the efficacy of GutGard (root extract of *Glycyrrhiza glabra*) in the management of *Helicobacter pylori* (*H. pylori*) gastric load. Participants diagnosed with *H. pylori* infection were randomly assigned to two groups to orally receive 150 mg of GutGard (*n* = 55) or placebo (*n* = 52) once daily for 60 days. *H. pylori* infection was assessed using ^13^C-urea breath test (^13^C-UBT) at days 0, 30, and 60. Stool Antigen test (HpSA) was also performed on days 0, 30, and 60. Repeated measures of analysis of variance (RMANOVA), chi-square, and Fisher's exact probability tests were used to compare the treatment outcomes. A significant interaction effect between group and time (*P* = 0.00) and significant difference in mean Delta Over Baseline (DOB) values between GutGard (*n* = 50) and placebo (*n* = 50) treated groups after intervention period were observed. On day 60, the results of HpSA test were negative in 28 subjects (56%) in GutGard treated group whereas in placebo treated group only 2 subjects (4%) showed negative response; the difference between the groups was statistically significant. On day 60, the results of ^13^C-UBT were negative in 24 (48%) in GutGard treated group and the difference between the groups was statistically significant. The findings suggest GutGard is effective in the management of *H. pylori*.

## 1. Introduction

*Helicobacter pylori* (*H. pylori*) is a gram-negative spiral, or helical shaped bacteria inhabiting the gastric epithelial cells [1], of half the world human population, with prevalence rates reported to be highly variable in different regions of the industrialized and developing countries ranging from nearly 7% to greater than 95% [2]. Presence of *H. pylori *is associated with an increased risk of developing upper gastrointestinal tract diseases, namely, peptic ulcer disease [3–5], gastric carcinoma [6, 7], and gastric MALT lymphoma. Also, World Health Organization classified *H. pylori* as a type I carcinogen for gastric carcinoma [8, 9].

Maastricht III Consensus and American College of Gastroenterology recommended standard triple therapy (a proton pump inhibitor (PPI), clarithromycin, and amoxicillin/or metronidazole) and Bismuth-based quadruple therapy (Bismuth with PPI and two antibiotics) as first line treatments in subjects infected with *H. pylori* [10, 11]. However, the success rates of these therapies have not been very encouraging. Despite the large number of studies, identifying an optimal regimen for *H. pylori,* treatment still remains a challenging clinical problem. The primary cause for failure reported in systematic review and meta-analysis reports is *H. pylori* resistance to the antibiotics [12, 13]. Although use of molecular test systems can detect the resistance, this does not provide long term solution to rising tendency of resistance to antibiotics [14, 15]. Besides resistance, adverse effects and poor patient compliance limit the efficacy of these regimens. Considering the limitations in treatment regimens, development of alternative remedies remains constant need. With the growing popularity for naturally occurring medicinal plants, herbal preparations have been evaluated for the management of *H. pylori *and one such medicinal plant that holds promise for *H. pylori* management is licorice [16]. Licorice (*Glycyrrhiza glabra* Linn; Family: Leguminosae) has been in traditional use for several centuries. The roots and rhizomes of *G. glabra* have been reported for antipyretic, antimicrobial, hepatoprotective, antioxidant, antiadhesive, anxiolytic, expectorant, laxative, and diuretic properties [17–20]. In addition *G. glabra* has antiviral, antiinflammatory, anticancer, anti-ulcer activities [21, 22].

*G. glabra* was reported to exhibit antimicrobial activity against several gram-negative and gram-positive bacterial strains including *H. pylori* [23]. Besides these, licorice also demonstrated beneficial effects on *H. pylori* through its antiadhesive properties [20]. Activity against ulcer and cancer, clinical outcomes of *H. pylori* infection were also exhibited by licorice. Curative effect of deglycyrrhizinated licorice (DGL) on ulcer has been reported *in vivo* and in clinical studies [24–26], whereas, anti-cancer effect of licorice extract was established in *in vitro* study [27].

GutGard is a deglycyrrhizinated root extract of *G. glabra*, the safety and efficacy of which was evaluated in several studies conducted earlier. *In vitro* battery of genotoxicity tests showed no evidence of clastogenic and mutagenic effects and in acute oral toxicity study GutGard was found to be safe up to 5000 mg/kg rat body weight [28]. A randomized, double-blind, placebo-controlled clinical study reported significant decrease in symptoms scores of functional dyspepsia and also did not report any major trial related adverse effects [29]. Furthermore, GutGard exhibited anti-inflammatory activity likely through inhibition of COX and LOX pathways [28] and anti-ulcer activity was demonstrated in pylorus ligation, cold-restraint stress, and indomethacin induced ulcer in albino Wistar rats in which at 12.5, 25, and 50 mg/kg dose levels, the effects were found in dose dependent manner [30]. 

From the above considerations *G. glabra* is found to have potential activity against gastrointestinal related disorders and this study in particular was aimed to assess the efficacy of GutGard, in the management of *H. pylori *in a randomized double blind placebo controlled trial.

## 2. Methods

### 2.1. Subjects

Subjects, aged between 18–45 years with positive response in *H. pylori* stool antigen test (HpSA) and ^13^C-urea breath test (^13^C-UBT), were enrolled. Subjects were excluded if they (i) had history of bleeding duodenal ulcer, MALT lymphoma, gastroesophageal reflux, surgery for ulcers; (ii) had advanced chronic illness, mental illness, dementia, or suffering with concomitant symptoms of the irritable bowel syndrome, (iii) were first level relatives to gastric cancer patients, (iv) were taking antibiotics and/or PPIs and/or H 2 -antagonists 2 weeks prior to the administration of the investigational product and were using nonsteroidal anti-inflammatory drugs, steroids, bismuth preparation, (v) were participating in other clinical trials, (vi) were pregnant/lactating, (vii) were engaged in drug or alcohol abuse.

### 2.2. Study Intervention

Each capsule of GutGard contains 150 mg of actives of *G. glabra*. GutGard is a flavonoid rich, root extract of *G. glabra* developed by Natural Remedies, Bangalore, India. GutGard has the following phytochemical specifications, namely, glabridin (≥3.5% w/w), glabrol (≥0.5% w/w), eicosanyl caffeate (≥0.1% w/w), docosyl caffeate (≥0.1% w/w), glycyrrhizin (≤0.5% w/w), and total flavonoids (≥10% w/w). 

### 2.3. Study Protocol

The double blind placebo controlled trial was conducted in D2L Pharma Research Centre, Bangalore, Karnataka, India, from July 2011 to November 2011. Ethics Committee approval was obtained for the conduct of the trial. A total of 215 subjects were screened and 107 subjects with positive response to HpSA test and ^13^C-UBT were recruited. The investigator clearly explained the purpose and methodology of the clinical trial in a simple, explicable language before taking consent from the subjects for participation in the trial. In addition the queries/doubts of trial subjects if any were clarified by the investigator prior to signing the consent form. The subjects were asked to completely understand and sign the informed consent form. The subjects were informed that they can withdraw from the study at any point without any prior notice. It was informed, if the subject volunteers to provide the reasons for opting out, consent to use this information will be taken from the subject. Following the consent, the subjects were randomly assigned to GutGard (*n* = 55) and placebo (*n* = 52) groups. A computer aided programme was used to generate randomization list and the random numbers were considered as subject code. As per the random allocation sequence, the containers (either GutGard or placebo) were labeled with unique random numbers. The entire process was carried out in a confidential manner and all the study related personnel, namely, investigators, subjects, and other supportive staff were unaware of the random allocation sequence. 

The study medication was dispensed by the pharmacist to the subjects taking into consideration the order of enrollment and as per the random allocation sequence. Both GutGard and placebo capsules were similar in appearance (size, shape, and color) and flavor including packaging. The study interventions were packaged and labeled identically to maintain blinding. The personnel (investigator, pharmacist, and subjects) involved in the trial were blinded during the trial period. Each subject was given a container of 30 capsules and was advised to take one capsule daily with a glass of water before food in the morning for 30 days. The subjects were informed to visit the trial centre on day 30 along with the container and the study diary card. The compliance to study medication was recorded by counting the leftover capsules in the container and from the diary card. After recording the compliance, another container of 30 capsules was provided to the subjects and the same procedure was followed at the scheduled followup on day 60.

### 2.4. Outcome Measures

The gastric load of *H. pylori* was assessed on days 0, 30, and 60. Decrease in *H. pylori* gastric load was assessed using ^13^C-UBT and HpSA test as outcome measures. The proportion of subjects with initial positive ^13^C-UBT and HpSA test results found to be negative at day 30 and day 60 was measured. 

### 2.5. Data Analysis

The required sample size for the GutGard clinical study in the management of *H. pylori* was calculated using the formula *n* = (8(*CV*)^2^/(PC)^2^)(1 + (1 − PC)^2^) [31] where proportionate change in means (PC) of 20% [32] with 35% of coefficient of variation (*CV*) was considered. Assuming a possibility of lost to followup or dropouts as 20% at least 50 subjects were needed for each group. Hence, the required sample size was calculated as 100 subjects for the entire study. 

At baseline, the characteristics of the subjects of the two groups were compared by independent sample *t*-test. The cure rates and the interaction effects between group and time were determined using per protocol (PP) analysis. The Delta Over Baseline (DOB) values were expressed as mean ± SD at days 0, 30, and 60 and were analyzed by repeated measures of analysis of variance (RMANOVA) and the statistical significance was set at *P* ≤ 0.05. Positive and negative responses from the HpSA test and ^13^C-UBT were assessed. The proportion of individuals showing positive and negative response to ^13^C-UBT in GutGard and placebo treated groups was analyzed using Fisher's exact probability test, and chi-square test was used to analyze the proportion of individuals showing positive and negative response to HpSA test. The statistical analysis of side effects was performed with the chi-square analysis. 

## 3. Results

**Figure 1:**
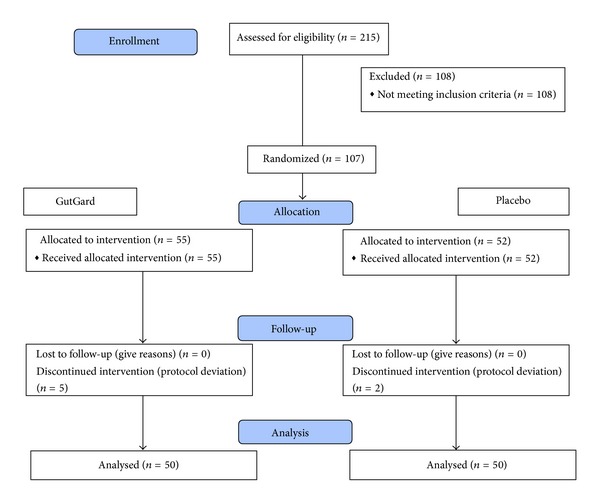
Flow chart of disposition of subjects.

A total of 215 subjects were screened initially, and 107 subjects were recruited. Seven out of the 107 enrolled were excluded from the study as they did not satisfy the inclusion criteria for age (subjects were over 45 years of age); finally, 100 subjects per protocol were analyzed (Figure 1). At baseline mean characteristics of treated group versus placebo were found to be comparable (Table 1). A significant interaction effect between group and time (df = 2,196; *F* = 1120.27; *P* = 0.00) and time effect was observed between the groups. Significant difference in mean DOB values was observed between GutGard and placebo treated groups after intervention period. The magnitude of decrease in the *H. pylori* load is summarized in Table 2. The proportion of subjects turned from positive to negative response status is elucidated in Table 3. At day 0 and day 30 all the subjects in placebo and GutGard treated groups showed positive response to HpSA test and ^13^C-UBT. On day 60, the results of HpSA test were negative in 28 subjects (56%) in GutGard treated group and 2 subjects (4%) in placebo treated group; the difference between the groups was statistically significant. On day 60, the results of ^13^C-UBT were negative in 24 (48%) in GutGard and one (2%) in placebo consumed subjects; the difference was statistically significant. 

*Safety*. Regarding the overall tolerability of interventions, in total 22 subjects (22%) showed at least one side-effect. One subject (1%) experienced moderate side-effect (fever); 21 subjects (21%) experienced mild side-effects, but none stopped the treatment and all have completed the study. The incidences of side-effects were considered to be not related to treatment. The profiles and frequencies of side-effects were listed in Table 4. On comparison, the frequencies of side-effects between GutGard and placebo treated groups were non-significant (Table 4).

## 4. Discussion

Extensive research in the past few decades since the discovery of *H. pylori* indicated that it is the major risk factor for gastrointestinal disorders and the research guidelines recommended that all *H. pylori* positive individuals be treated irrespective of the clinical outcome. The eradication of *H. pylori * in the infected subjects will not only prevent *H. pylori * associated diseases but also limit the spread of infection [33]. Albeit, different regimens are available for the treatment of *H. pylori*, the success rates of these regimens are low due to the rising prevalence of antimicrobial resistance and an effective regimen for *H. pylori* still remains elusive. Use of herbal supplements as alternative sources has attracted the researchers worldwide over the past few years and several studies on medicinal plants have been undertaken to evaluate the anti-*H. pylori* effects [34–37]. From the published preclinical studies, *G. glabra* is reported to possess activity against *H. pylori* [23, 38, 39]; however, the major concern is the validation of these effects in well designed clinical settings. In the present study, GutGard, an extract of *G. glabra*, has been evaluated in a double blind placebo controlled trial for its efficacy in the management of *H. pylori* representing one of the pioneering studies in this aspect.

In the present study, effectiveness of GutGard supplementation for 60 days was evaluated in subjects positive for *H. pylori* based on the HpSA and ^13^C-UBT results pre- and posttreatment. An interaction effect with significant difference in mean DOB values between GutGard and placebo treated groups after intervention period was observed. DOB is increasingly recognized as a quantitative measure of *H. pylori * gastric load [40]. The bacterial urease activity, which correlates with DOB values, mainly depends on the overall bacterial load [41] and some studies have suggested that high DOB values are associated with a high bacterial load in the stomach [42–44] as well as with *H. pylori* virulence factors, such as CagA [45, 46]. The data on GutGard indicates that the *H. pylori* load was significantly decreased in GutGard treated subjects as compared to placebo treatment. Apart from decrease in gastric load of *H. pylori*, the GutGard treated subjects showed negative response in ^13^C-UBT and tested negative in HpSA test. The results of HpSA and ^13^C-UBT in terms of number of subjects with negative *H. pylori * test findings are in concordance with earlier study outcomes which demonstrated that ^13^C-UBT and HpSA are absolutely equivalent in terms of sensitivity and specificity in the evaluation of eradication therapy [47–49].

Several studies have evaluated the effects of supplementation of extracts of medicinal herbs along with standard treatment regimens in the management of *H. pylori *[50, 51], and only few studies evaluated the effect of herbal preparations as a stand alone or along with antacids. Zhang et al. reported that 14.43% of the subjects evaluated in a double-blind randomized placebo-controlled trial showed negative results for *H. pylori* after 90 days of supplementation with Cranberry juice [32]. Administration of 5 g of vitamin C for 4 weeks in *H. pylori* positive patients with chronic gastritis resulted in recovery of 30% of the patients treated with vitamin C [52]. Treatment effects with 1 g, 2 g, and 3 g *Nigella sativa* administered with omeprazole were 47.6%, 66.7%, and 47.8% [53], respectively, while as a stand alone supplement GutGard showed 56% cure rate. Results of earlier clinical studies provide insights on the eradication rates of mono-, dual, and triple therapies. The eradication rates reported for monotherapy was 0–54%, and dual therapies revealed recovery rate of 50–85%. Further the triple therapies that are recommended as first line option were reported to have a cure rate of 95% [54, 55]. However, in actual clinical settings even the triple therapies have been reported to have shown eradication rates of less than 80% [56, 57]. The results of acid stable effective monotherapy for the treatment of *H. pylori * as a stand alone were comparable to GutGard cure rates. The fact that the eradication rates in clinical settings for antibiotic regimens are very low indicates preexisting resistance of *H. pylori* to antibiotics due to wide spread use of antibiotics for other indications, side effects, and premature discontinuation of antibiotic use. In such a scenario, GutGard that is well tolerated, safe, and with effective cure rates would be a better alternative for the management of *H. pylori*. As there are genetic differences in *H. pylori* strains in east and west [58], further research in different locations and investigating the effect of GutGard in subjects resistant to antibiotics, subjects with treatment failure to triple therapy, or evaluating effectiveness of GutGard in combination with proton pump inhibitors/other antibiotics as dual or triple therapy will further establish the effectiveness of GutGard.

The activity of GutGard on gastric *H. pylori* may be explained by various possible mechanisms. Based on the findings by Fukai et al. [38], about the anti-*H. pylori* activity of licorice, the anti-microbial activity of GutGard was investigated using *in vitro* assays such as DNA gyrase inhibition, protein synthesis inhibition, and dihydrofolate reductase (DHFR) enzyme inhibition. DNA gyrase is an essential bacterial enzyme that catalyzes the ATP-dependent negative supercoiling of double-stranded closed-circular DNA. DNA gyrase is vital for transcription and replication of bacteria [59]; inhibition of DNA gyrase appears to be an opt target for anti-microbials. GutGard has shown activity by inhibiting DNA gyrase [60]; the results are in accordance with the study published by Hui et al. [61]. Interestingly, GutGard also inhibited protein synthesis and DHFR enzyme *in vitro* [60]. Blockade of DHFR causes cell death through inhibition of DNA synthesis and is considered suitable target for inhibition of *H. pylori* replication [62]. The aforementioned mechanisms may attribute to the effect of GutGard on *H. pylori * management. 

GutGard was found to be safe and well tolerated. Few side effects, namely, nausea, diarrhoea, headache, throat pain, vomiting, cold and cough, body pain, acidity, body heat, fever, and pain in stomach were observed mostly in both placebo and GutGard treated groups. However, side-effects recorded did not reveal any significant differences between treatment groups and were found to be non-treatment-related. The published literature on clinical studies of licorice formulations also did not report any significant adverse events that indicate the safe nature of the dietary supplement [24]. The safety on present intake levels of GutGard is also affirmed in the study by Raveendra et al. [29]. 

## 5. Conclusion

In conclusion, the findings of the randomized double blind placebo controlled trial on GutGard, extract of *G. glabra* revealed significant decrease in the *H. pylori*, gastric load as compared to placebo and was found to be safe and well tolerated. In the present study, treatment with GutGard was found to be 73.2% or 3.73 times more effective than placebo. Hence GutGard supplementation can be considered an effective alternative remedy for the management of *H. pylori*.

## Figures and Tables

**Table 1:** Characteristics of the subjects at baseline (mean ± SD).

Parameters	GutGard (*n* = 50)	Placebo (*n* = 50)
Subjects (male/female)	23/27	23/27
Age (years)	32.86 ± 6.50	33.10 ± 5.59
Weight (kg)	62.60 ± 7.43	62.31 ± 7.79
Height (cms)	166.12 ± 8.45	165.56 ± 7.67
Heart rate/min	69.34 ± 4.30	69.08 ± 4.26
BP systolic (mmHg)	116.72 ± 6.44	115.40 ± 6.98
BP diastolic (mmHg)	80.80 ± 5.29	80.28 ± 4.43
HpSA	Positive	Positive
*δ* (means)	7.12 ± 1.36	6.88 ± 1.34

*P* ≤ 0.05 versus placebo.

*δ*: delta over baseline value of ^13^C urea breath test.

**Table 2:** Effect of GutGard on *H. pylori* gastric load (mean ± SD).

Parameter	Groups	Day 0	Day 30	Day 60	Significance			
					Variables	df	*F* value	*P* value
DOB	GutGard	7.12 ± 1.36	6.24 ± 1.24	4.21 ± 1.15	Group	1,98	5.63	0.02
	Placebo	6.88 ± 1.34	6.40 ± 1.31	6.10 ± 1.30	Time	2,196	3047.10	0.00
					Group × time	2,196	1120.27	0.00

**Table 3:** Proportion of subjects turned from *H. pylori*—positive (Hp^+^) to negative (Hp^−^) status as measured by HpSA and ^13^C-UBT.

Groups	Days	*n*	HpSA		^ 13^C-UBT	
			Hp^+^	Hp^−^ (%)	Hp^+^	Hp^−^ (%)
GutGard	0	50	50	0 (0)	50	0 (0)
Placebo		50	50	0 (0)	50	0 (0)
GutGard	30	50	50	0 (0)	50	0 (0)
Placebo		50	50	0 (0)	50	0 (0)
GutGard	60	50	22	28* (56)	26	24* (48)
Placebo		50	48	2 (4)	49	1 (2)

*n*: no. of subjects; Hp^−^: *H. pylori* negative; Hp^+^: *H. pylori* positive.

%: percentage of subjects turned from Hp Positive to Hp Negative.

*Significant difference compared to placebo.

**Table 4:** Side effects during intervention period.

Side effect	GutGard (*n* = 50) *n* (%)	Placebo (*n* = 50) *n* (%)	*P* value
Mild diarrhoea	5 (10)	2 (4)	0.24
Mild headache	1 (2)	1 (2)	1.00
Mild vomiting	3 (6)	1 (2)	0.30
Mild nausea	1 (2)	1 (2)	1.00
Mild throat pain	1 (2)	1 (2)	1.00
Mild cold and cough	1 (2)	1 (2)	1.00
Mild body pain	1 (2)	1 (2)	1.00
Mild fever	2 (4)	0	0.15
Moderate fever	1 (2)	0	0.31
Acidity	1 (2)	1 (2)	1.00
Mild pain in stomach	1 (2)	0	0.31
Mild body heat	1 (2)	3 (6)	0.30
